# The Law

**Published:** 1890-07

**Authors:** 


THE law to divorce the teaching from the licensing authority in this State has finally passed, after a seven years' struggle with the opposition. If its provisions are considerately and loyally sustained, we venture the opinion that it will prove satisfactory. If, on trial, it should show defects, these can easily be remedied; but no respectable physician, no decent college, no medical society of standing, no clique or coterie whatever, can afford to be found throwing obstacles in the way of this reform, or threatening to move for amendments to the law before it is even tried. Any such will at once take their place where they belong, viz., with the enemies of progress, for this is where all right-thinking people, who are always in numerical majority, will class them. Henry Watterson's "Star-eyed goddess" would not even deign to glance slyly at them.

